# Supplementary material for: Understanding the effects of stress on the P300 response during naturalistic simulation of heights exposure
Source: PLoS One. 2024 Apr 17;19(4):e0301052. doi: 10.1371/journal.pone.0301052 (PMC11023450; doi:10.1371/journal.pone.0301052)
Supplement: S1 Questionnaire — (PDF) [file pone.0301052.s001.pdf]

ID number:

Trial QUESTIONNAIRE (Done Verbally)

SAM diagram is shown in the VR paradigm

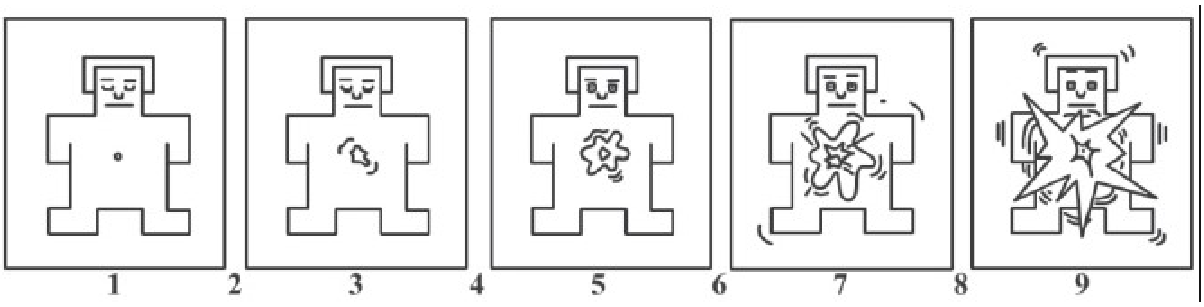

How would you rate your stress levels after that walk based on the diagram in front of you? Where 9 is extremely stress and 1 is not stressed at all.
1 2 3 4 5 6 7 8 9

T1	T2	T3	T4

T1-1	
T1-2	
T1-3	
T1-4	
T1-5	
T2-1	
T2-2	
T2-3	
T2-4	
T2-5	
T3-1	
T3-2	
T3-3	
T3-4	
T3-5	
T4-1	
T4-2	
T4-3	
T4-4	
T4-5	
