# Supplementary material for: Understanding the effects of stress on the P300 response during naturalistic simulation of heights exposure
Source: PLoS One. 2024 Apr 17;19(4):e0301052. doi: 10.1371/journal.pone.0301052 (PMC11023450; doi:10.1371/journal.pone.0301052)
Supplement: Attachment — Submitted filename: Response to Reviewers.pdf [file pone.0301052.s002.pdf]

Dear PLOS ONE Editorial team and reviewers,

Thank you for providing us with detailed and well-written reviews/comments to help improve our manuscript. My apologies for the delay in providing the revision; we required an extension due to health issues in the prior months. We've reviewed the comments from the editor and reviewers and revised many parts of the paper. We hope our revision has addressed the concerns and comments from the reviews. We've provided the labelled (highlighted revisions) and the unlabelled manuscript in the revision. In this document, we will respond to the specific points raised by the editor and reviewers and provide details on how the revision addresses the points.

Editor/Additional Requirements:

1. Please ensure that your manuscript meets PLOS ONE's style requirements, including those for file naming. The PLOS ONE style templates can be found at https://journals.plos.org/plosone/s/file?id=wjVg/PLOSOne_formatting_sample_main_body.pdf and https://journals.plos.org/plosone/s/file?id=ba62/PLOSOne_formatting_sample_title_authors_affiliations.pdf

We have modified our latex template to the PLOSOne template link on this website.

2. Please change "female" or "male" to "woman" or "man" as appropriate, when used as a noun (see for instance https://apastyle.apa.org/style-grammar-guidelines/bias-free-language/gender).

We have modified the nouns to the suggested more appropriate wording.

3. Please update your submission to use the PLOS LaTeX template. The template and more information on our requirements for LaTeX submissions can be found at http://journals.plos.org/plosone/s/latex.

Addressed in point 1.

4. Thank you for stating the following in the Acknowledgments Section of your manuscript:

"This work was supported in part by the Australian Research Council (ARC) under discovery grant DP210101093 and DP220100803. Research was also sponsored in part by the Australia Defence Innovation Hub under Contract No. P18-650825, US Office of Naval Research Global under Cooperative Agreement Number ONRG - NICOP - N62909-19-1-2058, and AFOSR – DST Australian Autonomy Initiative agreement ID10134. We also thank the NSW Defence Innovation Network and NSW State Government of Australia for financial support in part of this research through grant DINPP2019 S1-03/09 and PP21-22.03.02."

We note that you have provided funding information that is not currently declared in your Funding Statement. However, funding information should not appear in the Acknowledgments section or other areas of your manuscript. We will only publish funding information present in the Funding Statement section of the online submission form.

Please remove any funding-related text from the manuscript and let us know how you would like to update your Funding Statement. Currently, your Funding Statement reads as follows:

"This work was supported by Australian Research Council (ARC, https://www.arc.gov.au/) (DP210101093 and DP220100803), Australia Defence Innovation Hub (https://innovationhub.defence.gov.au/, P18-650825), US Office of Naval Research Global (https://www.nre.navy.mil/, ONRG - NICOP - N62909-19-1-2058), AFOSR – DST Australia Autonomy Initiative (ID10134), and NSW Defence Innovation Network (https://innovationhub.defence.gov.au/, DINPP2019 S1-03/09 and PP21-22.03.02) to Chin-Teng Lin. The funders had no role in study design, data collection and analysis, decision to publish, or preparation of the manuscript."

Please include your amended statements within your cover letter; we will change the online submission form on your behalf.

My apologies for the oversight in the listing of funding bodies. We have removed the funding information from the manuscript. This funding statement listed above looks correct and won't need to be updated:

"This work was supported by Australian Research Council (ARC, https://www.arc.gov.au/) (DP210101093 and DP220100803), Australia Defence Innovation Hub (https://innovationhub.defence.gov.au/, P18-650825), US Office of Naval Research Global (https://www.nre.navy.mil/, ONRG - NICOP - N62909-19-1-2058), AFOSR – DST Australia Autonomy Initiative (ID10134), and NSW Defence Innovation Network (https://innovationhub.defence.gov.au/, DINPP2019 S1-03/09 and PP21-22.03.02) to Chin-Teng Lin. The funders had no role in study design, data collection and analysis, decision to publish, or preparation of the manuscript."

5. In your Data Availability statement, you have not specified where the minimal data set underlying the results described in your manuscript can be found. PLOS defines a study's minimal data set as the underlying data used to reach the conclusions drawn in the manuscript and any additional data required to replicate the reported study findings in their entirety. All PLOS journals require that the minimal data set be made fully available. For more information about our data policy, please see http://journals.plos.org/plosone/s/data-availability.

Upon re-submitting your revised manuscript, please upload your study's minimal underlying data set as either Supporting Information files or to a stable, public repository and include the relevant URLs, DOIs, or accession numbers within your revised cover letter. For a list of acceptable repositories, please see http://journals.plos.org/plosone/s/data-availability#loc-recommended-repositories. Any potentially identifying patient information must be fully anonymized.

Important: If there are ethical or legal restrictions to sharing your data publicly, please explain these restrictions in detail. Please see our guidelines for more information on what we consider unacceptable restrictions to publicly sharing data: http://journals.plos.org/plosone/s/data-availability#loc-unacceptable-data-access-restrictions. Note that it is not acceptable for the authors to be the sole named individuals responsible for ensuring data access.

We will update your Data Availability statement to reflect the information you provide in your cover letter.

We have uploaded the data required to replicate the results outlined in the manuscript to a public repository (Dryad). Please let us know if there is any issue with accessing the data.

Repository link:
https://datadryad.org/stash/share/tRM7LgrL_ecB9ghA632P_FXbMIYN6Yo8121G1f36mak

6. Please include your full ethics statement in the 'Methods' section of your manuscript file. In your statement, please include the full name of the IRB or ethics committee who approved or waived your study, as well as whether or not you obtained informed written or verbal consent. If consent was waived for your study, please include this information in your statement as well.

We have updated the participant section in the methods to include the human research ethics committee (UTS), the approval ID, and the method of consent provided (informed and written).

Reviewer 1:

Reviewer #1: The experimental setup is interesting. However, this reviewer has some concerns which have to address during major revision.

Thank you for the comments and the interesting paper suggestions; we have learnt a lot from the papers and included the methodology in our analysis and manuscript; we've cited the paper in the areas where references are from the suggested papers.

1. The author should define "stress" in this context clearly. It would be better to replace "stress" by the word which can be measured quantitatively and/or having the standard questionnaire in identifying the level of mental or cognitive world load during interested periods. This famous related review article might help P. Consumer Grade EEG Measuring Sensors as Research Tools: A Review.

Thank you for pointing out the ambiguity in the definition of stress. We've added further clarification to the definition of physiological stress in the introduction (opening sentences). Specifically, we've outlined the Russell circumplex model of assessing stress through arousal and valence, which directly links to the SAM questionnaire used in this manuscript. These measures were also mentioned in the review article, so we included them in the introduction.

We are hesitant to replace the word "stress" in this manuscript because multiple past similar works outlined in Table 1 directly use the word stress and relate it to the P300 response. Since this work aims to further the previous works listed directly, it would be more appropriate for the manuscript to follow the terminology and methodology established in the previous literature.

2. P300 or ERSP visualization and analysis need further improvement and exploration. The recent work with patients might help the author in the improvement of this point. This reviewer suggest the author to study more from A Pilot Study on Visually Stimulated Cognitive Tasks for EEG-Based

Dementia Recognition.

The suggested paper is very relevant and helpful in revising the P300 and ERSP results. We've reworked our data analysis, and figures 8, 10, and 11 now follow closely with the methodology and presentation of the suggested paper. We've referenced the paper in our methodology to indicate where we followed the outlined method.

3. Also the analysis of EEG to together with response or reaction times can learn from: Supervision of a self-driving vehicle unmasks latent sleepiness relative to manually controlled driving.

This suggested paper was very helpful in improving our reaction time results. We followed the reaction time analysis outlined in the paper and incorporated figure 3 to show better the distribution of the participant's change in reaction time.

Looking forward to reading the revised version.

Thank you for the great paper suggestions; we hope we have appropriately addressed the concerns from the review.

Reviewer 2

Reviewer #2: This is an interesting study on P300 responses under real and virtual height exposure. Basically, the experimental design is somewhat unbalanced (and hard to understand from the description) but it's a good approach towards a quite timely question. And this is the main critique from my side. This is a nice pilot for a completely controlled study, but, in particular with some minor faults in the design and a sample size of 17 that additionally is divided into two groups, this is not sufficient for a publication. Along with this criticism, it should be noted that the main effect (the modulation of P3 amplitude in one of the two groups) just touches significance ($Z=1.96$, $p=0.049$) although the authors seem to have renounced to correct their data against alpha inflation.
As the authors claim in the final section, they were kind of surprised by the interindividual differences in behavior.

Thank you for the detailed comments in the review; we highly appreciate the effort and thought into the feedback. We've revised our data analysis methodology and results to address your concerns with the manuscript, hopefully. Specifically, with the significance, we've revised the test to include post hoc analysis to produce more rigorous results.

Those are, besides the fact that mobile EEG is used in order to investigate more realistic behavior, an interesting point of this study, however, the authors nowhere provide evidence that there are really two distinct groups. They simply divided their sample by a split half on one RT parameter, which was for sure not bimodally distributed. The reader might have the impression that they searched for significance based on different analyses strategies and this one turned out to provide a p value below .05.

We've revised the division section to clarify the motivation behind splitting our sample group. We've also added figure 3, which better depicts the spread of difference in performance between the ground and heights condition. The main reason we chose this categorization is that (as shown in

figure 3) is that some participants performed better in the high-stress condition (hence the negative difference between PH and GG). Hopefully, the revised text and figure 3 can clarify why we split the sample.

The entire MS appears to be intended in another context as presented. The introduction tlks a lot about BCIs, which are nowhere touched experimentally. This topic does not return before the final conclusion. The entire discussion section focuses on stress and cognition, which in my mind is the central issue here.

My apologies for the confusion/ambiguity with the mention of BCI. The inclusion of BCI was mainly due to differing opinions among the authors, as we wanted to include BCI as a higher-level motivation. We fully agree with your point that stress and cognition are the project's primary focus and key aim (investigating the effects of stress on cognitive performance). We've modified the introduction and methodology to be better focused on stress and cognition rather than BCI.

Minors

• The P300 paradigm is poorly described and not a standard for the P300 literature. Maybe this approach is usual in connection with PCIs, but the majority of visual oddball tasks uses much shorter stimulus presentations, longer interstimulus intervals which are normally additionally jittered.

Thank you for pointing out this issue; we have added more literature and rationale to the section. The trial structure contained elements of rapid serial visual presentation (rsvp) task design which is common for BCI research. We kept it named an oddball task mainly due to the timing and structure being closer to an oddball than an rsvp (as the stimuli are not rapidly presented). Please let us know if you feel the name should be changed.

• Why was the SAM presented before the trials? Would not a survey after performing the task better reflect stress?

We mainly chose to show the SAM before the start of the trials and during rests to minimize delay during the walking trials (participants would verbally rate the SAM score after each trial/walk). In hindsight, it would have been better to have visualized the SAM within the VR.

• Removing any component that is less than 85% classification for brain component (done with ICLabel?), is a hard criterion. How many ICs remained in the data then?

Thank you for pointing out this issue; we have revised our methodology, as 85% was a high criterion. We revised the preprocessing based on Makoto's pipeline (from sccn). The revised process considers the brain classification as the dominant label rather than a thresholded percentage. We also check for residual variance and dipole location.

• The authors appear to make separate tests for within group effecs and between groups effects on the same parameter. Was any correction against alpha error inflation applied?

We've revised the statistical test to now test for group effects. We applied a Friedman test (suggested by R3) and post hoc analysis through the Tukey HSD.

• statistical parameters are missing in many places. Also not significant effects should be presented with their numbers.

Thank you for pointing out this issue; we have revised the statistics and ensured consistent reporting. We've also added parameter reporting for when no significant effect was observed. Please let us know if we missed any specific parameter/section.

• Another evidence for effect searching: In the introduction they speak about Alpha and Theta ("276 pected P300 ERSP response is the alpha and theta synchronization during the onset (before) of the P300 peak and a gradual beta desynchronization during the P300 peak"), in the result section they present an effect in Beta power, and in the conclusion they added gamma activity. When I look at the time frequency plots, I rather see Mu responses in Group 2, which might reflect encapsulation due to the balance task.

My apologies for the confusing text, that line in the methodology refers to the background literature, which describes the characteristics of a P300 response in the ERSP. The results/conclusion reported on the findings from the data. We've fixed the section by adding the appropriate references to clarify the statement drawn from the literature.

Reviewer 3:

Reviewer #3: This study tries to address the influence of stress under a naturalistic environment on the P300 response. This research is very practical, and the paradigm design is also very interesting. However, many critical problems of statistical analysis in this manuscript make the results of this study unconvincing. Carefully checking the processes of statistical analysis and data processing are necessary before resubmitting the manuscript.

Thank you for the thorough review and detailed comments; we've used your feedback to try to fix and revise the statistical analysis and reporting of the methodology.

Major concerns:

1. In the pipeline of EEG preprocessing, the CAR can't be done before the ICA. As warned by the tutorial of the EEGLab and the theory of ICA, the common averaged reference will reduce the rank of data. The sequence of preprocessing processes needs to be carefully examined.

We agree that performing CAR reduces the data's rank and can be an issue if the number of ranks is equal to the number of channels. We should have (my apologies) mentioned that our EEG system used a reference electrode during the recording. So, the number of channels is 64 active electrodes + 1 FCz reference electrode. Before applying CAR, we add the reference electrode to the data. This action is equivalent to the "Add current reference channel back to the data "in the re-reference function by EEGLab. This way, when CAR is applied, we would have a rank of 64 equal to the number of probe electrodes and IC components after applying ICA. The code used below:

```
CleanEEG.nbchan = CleanEEG.nbchan+1;
CleanEEG.data(end+1,:) = zeros(1, CleanEEG.pnts);
CleanEEG.chanlocs(1,CleanEEG.nbchan).labels = 'initialReference';
CleanEEG = pop_reref(CleanEEG, []);
CleanEEG = pop_select( CleanEEG,'nochannel',{'initialReference'});
CleanEEG = eeg_checkset(CleanEEG);
```

This issue was outlined in Makoto Miyakoshi's blog/forum site:
https://sccn.ucsd.edu/wiki/Makoto's_preprocessing_pipeline

In -> 8. Re-reference the data to average -> What is rank?

Do, T. T. N., Lin, C. T., & Gramann, K. (2021). Human brain dynamics in active spatial navigation. Scientific Reports, 11(1), 1-12.

We have revised the preprocessing to clarify this issue, and the reference electrode was added back to avoid the rank issue.

2. What is the reference for choosing the time interval from 300ms to the reaction time for peak amplitude extraction? Please provide a further explanation of the temporal analysis window.

For the P300 peak detection, we chose the window 300-500ms based on the literature/previous papers on P300 peak detection. We've revised the methodology to clarify better why we chose the temporal analysis window and cite the reference we used.

3. Please give a further illustration of the "median split" of dividing participants into two groups. Moreover, as the split is performed "for difference in RT between GG and PH", this approach will increase the type I error of the following statistical analysis. If such a split is necessary, the split based on objective conditions such as baseline conditions is more acceptable.

We've revised the section to clarify better how and why we performed the median split. We also provided a figure that shows the individual participant's RT result (as requested in point 8).

4. In the paradigm design, four tasks, GG, PG, PP, and PH, are set. Why are there no GP and GH tasks?

The main reason is time, we originally had all 6 conditions, but our pilot experiments were around 3-4 hours. Each condition takes around 20-30 minutes. Our setup was quite complex as we had many safety protocols for walking on the platform, as it involved a safety harness and had fall risks. It would not have been feasible to perform all 6 conditions in a single session, and most participants were unwilling to come to multiple sessions (will need to repeat the setup). We chose to include GG, PP, and PH as the intuitive choice for three levels of height. The main choice was between PG, GP, and GH. The main novelty of the experiment was the walking platform, so we chose PG to explore the walking platform further. We reasoned that GP and GH were well documented in the literature, while we have not seen any condition where a person is physically elevated but virtually on the ground.

5. In addition, how is the order of the actual scene and the virtual scene determined? Why is the actual scene placed before the virtual scene? Or in other words, why use PG instead of GP?

My apologies if I misunderstand this question; the participant is wearing VR for all the conditions, so the virtual and actual scenes are played simultaneously, and the environment is mapped through optical motion capture.

If the question is why we chose PG over GP, it is mainly due to the reason mentioned in point 4: the physical platform was the main novelty. We wanted to include more conditions on the physical platform.

6. There should be four kinds of experiments in Randomized Walking Conditions in Figure 2, and only 1 kind of GG is drawn in this figure.

My apologies for the oversight in figure 2. Thank you for pointing this out. We've corrected the figure to include the four conditions.

7. The authors need to supplement the questionnaire used by SAM. Was this questionnaire filled out before and after the experiment or just after the experiment for each condition?

Thank you for the reminder; we will attach the questionnaire sheet to the revision submission. The participants verbally provided a SAM score after each walk.

8. The authors divided the subjects into 2 groups according to RT. Please add the RT result of each subject.

Based on your suggestion, we have added Figure 3, which presents the individual RT results to clarify how better/why we split the sample.

9. The authors conducted a correlation analysis between SAM and RT, but this paper wants to study the relationship between Physiological Stress and P300. Why do you not perform a correlation analysis between SAM and the amplitude or power of P300 in the two groups of subjects?

Thank you for the suggestion; based on this, we added Figure 9 and an additional result section for the correlation between SAM and P300 peak amplitude.

10. It can be seen from Figure 6 that Pz is not the strongest in the EEG scalp map of Group 2, so is it reasonable to use only Pz to calculate the P300 amplitude in Figure 7?

We've revised the topography results based on EEG analysis methodology changes. The new topography result seems consistent with Pz being within the activation region. We also want to keep consistent past stress, and P300 works referenced in table 1 feature Pz in their analysis.

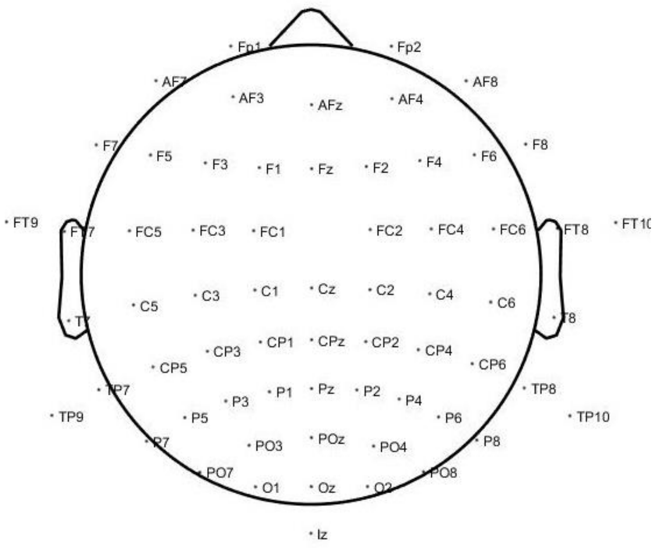

11. There are many problems in the statistical test. First, a major problem is using the paired test for multi-level comparison. As mentioned in the manuscript, "the RT, SAM, P3 peak and frequency power band data were not normally distributed", and some non-parameter methods like the Friedman test can be used to determine whether multiple conditions are significantly different. The paired test can not determine the effect of a factor with multi-level. This problem influences the statistical test of RT, SAM, ERP amplitude, and the comparison between two groups. Second, the correction of the p-value is ignored when multi-comparisons are made. The manuscript doesn't give an illustration of the correction method when RT, SAM, and ERSP are compared among four conditions (i.e. GG, PG, PP, and PH).

Based on your feedback, we've revised the statistical analysis in the methodology to the Friedman Test with Post Hoc Tukey HSD for condition comparison.

Third, the objects of statistical analysis are not uniform. In some analyses, the object is the mean value of each subject, while in other analyses, the object is the value of each trial. Please choose a uniform object and ensure that the object meets the assumptions of the statistical test method.

We've also revised the results by removing the trial/repeated measured analysis and having all the measurements uniform to the mean value by subject.

5. The specific details of ERSP need further explanation. For example, the method of doing time-frequency decomposition, the window length of ERSP, the baseline correction method of ERSP, and the epoch range of data used by ERSP are needed to determine the effectiveness of ERSP results.

We've revised the ERSP section to specify the steps involved in feature extraction, as outlined in this point.

Minor issues

1. On page8 line 299, there should be repeated measures ANOVA instead of "repeated measure ANOVA".

This line was removed after the revision to the statistical method. No longer using the repeated measure.

Thank you for your feedback; please let me know if you have any additional comments or feedback.

Kind regards,

Howe
