# Supplementary material for: Understanding the effects of stress on the P300 response during naturalistic simulation of heights exposure
Source: PLoS One. 2024 Apr 17;19(4):e0301052. doi: 10.1371/journal.pone.0301052 (PMC11023450; doi:10.1371/journal.pone.0301052)
Supplement: Attachment — Submitted filename: ResponsetoReview_2ndRevision_2023.pdf [file pone.0301052.s004.pdf]

Response for PONE-D-22-20226R1
Manuscript Title: Understanding the Effects of Physiological Stress on the P300 Response

Thank you for the detailed review from the reviewers and editors. I would like to apologise for the late response and revision. The extension was requested primarily due to health issues which delayed the revisions. We hope our revisions and response have addressed the concerns of the reviewers.

Reviewer 1
Thank you for the previous feedback and recommendations for improving our paper. We appreciate the time and thought spent on the reviewing process.

Reviewer 4
The study presented by the authors investigates the claimed relationship between physiological stress and cognitive function using a height-stressor task and a virtual reality scenario. An Oddball task was incorporated into the experimental scenario, and ERP measures taken to probe P300 response as a function of exposure to stress. Additional measures included Self Assessment Mannikin questionnaire to assess arousal level of the participant, and reaction times captured to stimuli in the oddball task. Results indicate significant differences in ratings of arousal on SAM but no significant differences based on RT or P300 ERP as a consequence of the experimental stressor exposure. Examination of data presented a basis for separating participants into two groups, differentiated by RT average between conditions – Group 1 performing worse when stressed, and Group 2 either unaffected or improving when stressed.

Principle points of criticism revolve around the definition of 'physiological stress' used, and the differentiation between groups based on the measures taken (including claims of a relationship between ability to cope with stress and the P300).

Firstly on the point of definition:
The title implies this pertains to 'physiological stress'. It is assumed throughout that the experimental scenario elicits physiological stress. Yet it is not totally clear whether this does in fact elicit physiological stress (as opposed to 'psychological stress' or *perceived* stress). This leads to a point concerning the use of the Self Assessment Mannikin as the avowed basis for determining measures of 'physiological stress'. Acknowledgement of the Russell-Circumplex model of valence-arousal is made, as a priori justification for using the SAM to measure (physiological) stress. Yet only the arousal component dimension of the SAM was measured, not valence and dominance. Is there a possibility that this is perhaps more indicative of 'perceived stress', based on an assumption here that 'arousal' and stress amount to the same thing. Typically when discussing physiological stress and measuring this in a behavioural study one might expect there to be more direct measures of, for instance, autonomic nervous system activity (eg. Heart rate variability). Such measures would more unequivocally determine that physiological stress is evoked within the experimental scenario. [Choi et al., (2017). Is heart rate variability (HRV) an adequate tool for evaluating human emotions? A focus on the use of the International Affective Picture System (IAPS). Psychiatry Res. 251:192-196. doi: 10.1016/j.psychres.2017.02.025. Epub 2017 Feb 11. PMID: 28213189.]

Given that such measures were not taken, at least qualifying the relationship to more directly physiological measures such as autonomic nervous system activity would provide a stronger case for using the SAM measures (and subsequent classification of individual or group differences). This includes reasoning for only focusing on the arousal dimension, not the other components of the circumplex model.

Thank you for the detailed feedback on the definitions used in the paper. We agree that the definition of physiological stress would not be adequately validated by a questionnaire and without physiological measures. As recommended, we have reworded the paper to remove the physiological component, as we do not have enough measurements to validate it.

Unfortunately, we were unable to collect the valance and dominance components. In hindsight, these metrics would have been useful. We only collected the arousal component due to the time constraints of the experiment. We selected arousal as prior literature suggested it tied closest to threat perception. We have revised the paper with additional reasoning/clarification for the selection of arousal and the exclusion of valance/dominance.

Group differentiation:

Participants were split into two groups based on their RTs, but RTs are likely influenced by a number of factors beyond stress levels, such as attention and impulsivity. Why not split participants based on SAM score or another measure of stress? The authors also claim that the experiment is aimed at investigating the relationship between the ability to cope with stress and the P300. However, the ability to cope with stress was not a variable in this study. Although the SAM results correlated with P300 amplitude across groups, when the groups were split, no effects were observed. This goes against the authors' claims that the difference between groups is a result of different abilities to cope with stress. As a suggestion, if focusing more on the relevance of SAM to unpack this (and split groups on this basis), the other dimensions of valence and dominance might usefully have contributed insight to ability to cope with the stressor. This includes understanding individual reactivity based on positive or negative valence of the experience and sense of control which may have bearing on their experience of the situation. [Jerram et al. (2013). The neural correlates of the dominance dimension of emotion. Psychiatry Res. 28;221(2):135-41. doi: 10.1016/j.pscychresns.2013.11.007. Epub 2013 Dec 4. PMID: 24359970.]

We agree that the SAM could reveal interesting metrics. Unfortunately, we could not adequately analyse this direction without the valance and dominance. Our intention with the SAM/arousal ratings was to provide some indication/validation that the height exposure elicits a stress response. The other factor is that the participant's arousal ratings were similar in that every participant's highest-rated condition was the PH condition, with a significant difference between GG and PH. Splitting solely based on arousal would result in two groups of similar ratings. In contrast, our participant RT distribution is more indicative of a difference between the two groups. In future studies, we will use the full SAM rating and physiological metrics to better investigate the difference in stress levels.

Trials with incorrect or untimely reactions were filtered out. It would be useful to know if there was a difference in the number of incorrect responses between groups. There may be a speed-accuracy tradeoff that is not accounted for when splitting the participants based on RT. How many trials were included in the analysis after incorrect trials and artifacts were removed? This can influence the EEG results obtained.

Thank you for highlighting this point. We have now included the trial distributions within the methodology section. We included both the distribution for all participants and the split groups. Overall, we retained a good trial distribution per participant. When comparing the GG and PH conditions (see table) we used for the group split; we do see a difference (not statistically significant) in Group 1 and Group 2 performance.

Group - Conditions	GG	PH
Group 1	M 83.89/100 SD 22.41	M 86.50/100 SD 19.75
Group 2	M 85.85/100 SD 17.25	M 94.50/100 SD 3.07

By referencing prior studies, the authors justify the election of the electrode of interest and the time window of interest for the P300. Even very small alterations in protocols can affect the timing and topography of the P300. As such, the electrode(s) and time window of interest should be defined by data-driven approaches such as PCA.

We understand the concern highlighted and agree PCA is a good approach to the methodology. As Table 1 of the paper mentions, our study builds on multiple prior studies that directly investigate the relationship between stress and P300. While our study does not directly recreate these works (only three selected oddballs and our study are the only ones to use height exposure for stress elicitation), our feature extraction method closely follows the prior studies. I've outlined the specific methodologies used in Table 1 (in the paper) studies in the table below. As observed, most prior works in this area select the p300 time window and electrode selection through literature, visual inspection of the ERP, or the grand average topography. We do not want to discount using PCA, but our methodology is more closely aligned with the methods of the prior studies for comparing stress and P300 behaviour. Most of the papers referenced are from reputable journals and have been published in the last 2-6 years. We have revised the methodology wording to follow the wording of the prior methods better.

Prior Study	Method of P300 extraction

Kamp et al. [1] 2021	Selected 300−500 ms and F3, Fz, F4, C3, Cz, C4, P3, Pz, P4 based on prior literature then selected Fz and Pz ERP based on visual inspection.
Ceballos et al. [2] 2017	Selected 350-560 ms and parietal electrodes based on prior literature.
Granovsky et al. [3] 1998	Selected 280–600 ms and Pz channel. Did not use literature (this paper is quite old) but selected channel (likely visual) from grand erp averages.
Dierolf et al. [4] 2017	Selected 300-370 ms based on prior literature and visually inspected the grand average topography for frontal and parietal channel (didn't explicitly state channel label) selection.
Jiang et al. [5] 2017	Selected 250–600 ms and Fz, FCz, and Cz based on inspection.
Mingming et al. [6] 2018	Selected 350 – 500 ms and FCz, Cz, and Pz channels based on prior literature sources and confirmed with grand average topographical maps.
Dierolf et al. [7] 2018	Selected 325–475ms and Fz, FCz, Cz, and Pz based on inspection (likely visual) of the grand average ERP for electrode.
Mingming et al. [8] 2020	Selected 450–600ms and Fz from preliminary inspection (likely visual) and stated it was consistent with prior literature.
Garcia et al. [9] 2019	Processed through UMA-BCI software classifier.

[1] https://doi.org/10.1016/j.biopsycho.2021.108095
[2] https://doi.org/10.15288/jsad.2012.73.761.
[3] https://doi.org/10.1016/S0168-5597(98)00036-7
[4] https://doi.org/10.1111/psyp.12826
[5] https://doi.org/10.1038/s41598-017-14138-w
[6] https://doi.org/10.1007/s00221-018-5283-6
[7] https://doi.org/10.1016/j.neuropsychologia.2018.08.020
[8] https://doi.org/10.1111/psyp.13521
[9] https://doi.org/10.1007/978-3-030-20521-8_37

With such a small sample size, individual differences or unknown variables preclude effects (hence why P300 amplitude correlates with SAM score across participants, but not when the groups are split).

This is an interesting study that seeks to extend the capacity to assess individual reactivity to stress and neural correlates thereof in a more ecological setting. On the basis of the comments outlined, however, and concurring with previous reviewer comments this might serve best as a pilot for a more comprehensive study that addresses the points raised. It is certainly recommended that the title either be changed to remove reference to 'physiological stress' or indeed qualify more clearly the link to physiological bases of the stress response.

Thank you for the time and effort spent on providing a comprehensive review. We agree with the points and have revised the manuscript based on your suggestion.
