# Supplementary material for: Understanding the effects of stress on the P300 response during naturalistic simulation of heights exposure
Source: PLoS One. 2024 Apr 17;19(4):e0301052. doi: 10.1371/journal.pone.0301052 (PMC11023450; doi:10.1371/journal.pone.0301052)
Supplement: Attachment — Submitted filename: ResponsetoReview_3ndRevision_2023.pdf [file pone.0301052.s005.pdf]

Response for PONE-D-22-20226R1
Manuscript Title: Understanding the Effects of Stress on the P300 Response During a Naturalistic Heights Exposure Simulation

Thank you for the time the reviewers and editors took to review and provide feedback to improve this manuscript. We hope our revisions and response have addressed the concerns of the reviewers.

Reviewer 4
We highly appreciate the time and effort taken to review our manuscript. Thank you for recognising the strengths and weaknesses of this manuscript and providing clear suggestions for improving this manuscript. We hope our revisions and response have addressed the concerns of the reviewers.

The authors have resubmitted the manuscript: “Understanding the Effects of Stress on the P300 Response” following reviewer feedback that raised some concerns based on the previous draft for submission. A primary issue highlighted previously centred on the title and contention that the study was evaluating physiological aspects of stress in the experimental scenario employed. Questions were also raised in relation to the Self Assessment Mannikin measures used: specifically only measuring the arousal dimensions (and not the valence and dominance components of the SAM). Objections were raised as to whether the experimental set-up was investigating physiological or psychological aspects of stress, and that by only capturing data on the arousal dimension, this might provide an incomplete picture of the model of stress being alluded to. Further comments questioned claims regarding ability to cope with stress and P300, despite no effects being observed when groups were split.

The authors have addressed key points including specific allusions to ‘physiological’ stress, removing reference to this. They also explained that the logistics of the experimental scenario meant that incorporating all of the dimensions of the SAM would have interfered with the naturalistic context in which the participant was situated. They provide a rationale on why the arousal dimension is valid to include as a measure in itself, as this has been shown to have a close relationship to threat perception. This reviewer recognizes the unique challenge that exists when running experiments that preserve a naturalistic context in which to elicit behaviours of interest that occur in real world situations, whilst also serving to employ robust measures that are usually collected in more contrived laboratory scenarios. This addresses the concerns around definitions of stress and the reasoning for using the subjective measures (SAM).

However, with respect to the analysis and assertions made about how the P300 relates to ability to cope with stress, the authors were unable to split participants based on a marker of stress. Rather, the split between groups was done on the basis of reaction times. As mentioned previously, splitting by reaction times can involve confounds unrelated to stress.

This should be acknowledged in the manuscript, or risk overstating claims made. Whilst the authors now provide the distribution of trials, it remains unclear if any speed-accuracy trade-off was apparent, or if there were any statistical differences between groups regarding error rates.

On the basis of the points addressed but with respect to the basis for splitting the data, it is recommended that minor revisions of the manuscript are made, modifying the discussion and conclusions to reflect the possibility that differences due to the split on RTs may not be specifically due to stress, and downplaying claims made about markers of ability to cope with stress. Such minor revisions would in this reviewer's view satisfy criteria for publication.

Thank you for your understanding and clear suggestion for the minor revision. We have revised the language to reflect better the claims made in this manuscript.

We have revised the methodology, discussion, and conclusion to outline the potential confounding factors of the splitting methodology. We highlighted that other factors may confound the results and reworded the claims to include the possibility that these factors can affect the results.

We have revised the abstract, introduction, discussion, and conclusion by rewording the statements on measuring the participant's ability to cope with stress. As outlined by the reviewer, we agree that this claim would be difficult to justify with the measurements collected in this study. We have revised the text to reflect the more exploratory aspect of understanding the P300 response when stressed rather than devising a new metric.

One additional suggestion concerns the title. It may be useful to emphasise the nature of the stressor/scenario from which the effects of stress are being derived, for specificity. For instance highlighting that this work is seeking to novelly extend research into the stress response/P300 into a more naturalistic environmental context, which is a particular strength of the work. This could prove fruitful for future lines of enquiry that differentiate 'stress' according to varied contexts beyond height exposure.

Thank you for this suggestion; we agree and have adjusted the title to include the aspects of naturalistic height exposure.
