# Supplementary material for: Understanding the effects of stress on the P300 response during naturalistic simulation of heights exposure
Source: PLoS One. 2024 Apr 17;19(4):e0301052. doi: 10.1371/journal.pone.0301052 (PMC11023450; doi:10.1371/journal.pone.0301052)
Supplement: Attachment — Submitted filename: ResponsetoReview_4thRevision_2024.pdf [file pone.0301052.s006.pdf]

Response for PONE-D-22-20226R1
Manuscript Title: Understanding the Effects of Stress on the P300 Response During a Naturalistic Heights Exposure Simulation

Happy New Year! Thank you for the time the reviewers and editors took to review and provide feedback to improve this manuscript. We hope our revisions and response have addressed the concerns of the reviewers.

Reviewer 4

The reviewer feedback concerning the split between groups has been more or less addressed with respect to potential confounds and limitations (eg. RTs perhaps being affected by factors other than 'stress' per se), including a reference to the accuracy scores. Although in the limitations section there is emphasis placed on RTs with respect to utility as a metric for gauging/estimating susceptibility to stress in conjunction with brain behaviour.

Other comments have been addressed from earlier revisions concerning definition of stress, although this reviewer still has some reservations about the qualification of stress in the experiment.

We highly appreciate the time and effort taken to review our manuscript. Thank you for providing clear suggestions for improving this manuscript. We hope our revisions and response have addressed the concerns of the reviewers.

"One group performed worse when stressed" - statements like this make presumption that a stress response has been elicited rather than the contention that the cognitive-affective-arousal state markers may be indicative of so-called 'stress' - this may sound like splitting hairs but nevertheless feels poignant to the argument being made in the paper about potential indicators of stress identified via the P300 and cognitive-arousal markers of performance...Indeed, the authors refer to 'stress levels' when they are talking about the different conditions. These would perhaps be more objectively described as 'potential stress-inducing conditions' - isn't the point of the experiment to examine whether, and to what degree, these conditions do actually elicit changes in 'stress levels'?

Thank you for highlighting your concern about using 'stress level' in this paper. We've reworded the paper to clarify the terming of stress better. The primary purpose of the experiment is to assess the behaviour of the P300 response when under stress. Walking at heights or height exposure is a recently popular stress elicitation paradigm, e.g. Peterson et al. (published in PLOS ONE). Based on our understanding and observation in the literature about height exposure and beam walking paradigms, stress is the most appropriate terminology for the paper.

Peterson, S.M., Furuichi, E. and Ferris, D.P., 2018. Effects of virtual reality high heights exposure during beam-walking on physiological stress and cognitive loading. PloS one, 13(7), p.e0200306.

Another point of contention rests in the defining of stress, in absolute, as a negatively valenced state. Whilst the authors reference the circumplex model, with stress generally associated with high arousal/negative valence, this generalisation does not reveal the whole picture. Further subdivision of stress into negative and positive dimensions (as is increasingly occurring in the literature – eg. Bak et al., 2022) - 'distress' and 'eustress' - paints a more complex and varied picture of stress. This may ultimately contribute to resolution of inconsistency in findings in the literature with respect for instance, to alternately, cognition-impairing and cognition-enhancing effects of exposure to 'stress'. It is a relevant point, particularly as research extends further into more naturalistic experimental scenarios territory, as the current study valiantly attempts. This is alluded to somewhat in the section: "Understanding Yerkes-Dodson Law and Participant's Performance when Stressed" in discussion of Group 2 vs Group 1 experiencing 'more optimal stress level'.

[Bak, S., Shin, J., Jeong, J. (2022). Subdividing Stress Groups into Eustress and Distress Groups Using Laterality Index Calculated from Brain Hemodynamic Response. Biosensors (Basel). 12(1):33.]

We agree that having valance measurements would have provided some interesting findings and potential to differentiate the participant experience further. Thank you for highlighting this interesting paper; we have revised the paper to include this work in our discussion.

"In summary, the two groups we found exhibited seemingly contradictory yet rational and supported by literature, P300 behaviours" - this sentence doesn't read clearly.

We've revised this sentence to improve the clarity.

Reviewer 5

This is a very interesting study, will contribute much for understanding the neural correlates of Stress. I appreciate this work on the idea and manipulations in the experiments, and also have some suggestions for clear state the findings and implications.

Thank you for your time and feedback. We hope that we have sufficiently answered your questions.

1. How to clearly distinguish the effect of emotional and stress during the procedure of experiment?

For the experimental paradigm, we are only considering the increase in arousal as the main indicator of stress. The emotional factor could play a small role in the response. However, the height exposure paradigm is primarily designed to evoke a physiological stress response. As observed in our SAM results, the paradigm was able to heighten the participant's arousal levels.

2. Is it possible to consider the link between stress and neural response in line with a curve model? That is, why participants performed better in some specific not all the conditions? How to reasonably to explain it?

Exploring a polynomial regression model could be interesting. In our discussion, we explored the Yorkes-Dodson model, a bell curve. Unfortunately, our correlation results do not have sufficient data points to apply a 2nd or 3rd-order polynomial regression without overfitting. Hence, our results only used linear regression.

Our discussions discuss potential reasons why certain participants performed better in some conditions. By splitting the participants, we observed a difference in EEG indicators, suggesting that certain participants were less able to focus when in the stressful conditions/

3. What's the meanness to discriminate the real and virtual environment?

We're not certain about the meaning of this question. Is there a specific section of the paper this question is referring to?

4. Did you control the daily stress and stress sensibilities in the data analysis?

We did not control for daily stress factors. Our work mainly follows the beam walking and height exposure virtual paradigms of Peterson et al. and Meehan et al. Exploring daily stress and stress susceptibility responses is less common in these exposure experiment designs. Height exposure is a reliable stress paradigm across various participant demographics. Short-term exposure can reliably elicit a response. This type of paradigm has become more popular among computational neuroscientists and behaviour psychological researchers due to the reliability and ease of set-up (due to Virtual reality).

Peterson, S.M., Furuichi, E. and Ferris, D.P., 2018. Effects of virtual reality high heights exposure during beam-walking on physiological stress and cognitive loading. PloS one, 13(7), p.e0200306.

Meehan, M., Insko, B., Whitton, M. and Brooks Jr, F.P., 2002. Physiological measures of presence in stressful virtual environments. Acm transactions on graphics (tog), 21(3), pp.645-652.

Examples of virtual height exposure paradigms

Kisker, J., Gruber, T. and Schöne, B., 2021. Behavioral realism and lifelike psychophysiological responses in virtual reality by the example of a height exposure. *Psychological research*, *85*, pp.68-81.

Fadeev, K.A., Smirnov, A.S., Zhigalova, O.P., Bazhina, P.S., Tumialis, A.V. and Golokhvast, K.S., 2020. Too real to be virtual: Autonomic and EEG responses to extreme stress scenarios in virtual reality. *Behavioural neurology*, *2020*.

Krupić, D., Žuro, B. and Corr, P.J., 2021. Anxiety and threat magnification in subjective and physiological responses of fear of heights induced by virtual reality. *Personality and Individual Differences*, *169*, p.109720.

Arachchige, S.N.K., Chander, H., Shojaei, A., Knight, A.C., Brown, C., Freeman, H.R. and Chen, C.C., 2024. Effects of virtual heights, dual-tasking, and training on static postural stability. *Applied Ergonomics*, *114*, p.104145.
