# Supplementary material for: Understanding the effects of stress on the P300 response during naturalistic simulation of heights exposure
Source: PLoS One. 2024 Apr 17;19(4):e0301052. doi: 10.1371/journal.pone.0301052 (PMC11023450; doi:10.1371/journal.pone.0301052)
Supplement: Attachment — Submitted filename: ResponseLetter.pdf [file pone.0301052.s007.pdf]

Response for PONE-D-22-20226R1
Manuscript Title: Understanding the Effects of Stress on the P300 Response During a Naturalistic Heights Exposure Simulation

Thank you for the time and effort you put into reviewing and significantly improving our manuscript. We hope our revisions and response have addressed the concerns of the reviewers.

Reviewer 5

Thanks for reviewing the paper again, the quality of the article has improved much. The authors answered the questions accurately and rigiously, I have one question that How to difine the virtual and real enviorment in the present study. How to exclude the effect of experiences from real environment in the virtual environment condition?

Response: We highly appreciate your suggestions, which improved our manuscript throughout the revision process.

In regards to the question, there are two instances concerning the virtual and physical/real-world environment. The first part is the local physical walking platform (Fig. 1.) in conjunction with the virtual environment. The second part is the paradigm being a more naturalistic real-world environment.

1. Physical and Virtual walking platform.

In our study, the physical and virtual environments work together to deliver an immersive height experience. Our work follows quite closely with several publications by Meehan et al. [1-3]. These publications are quite well-known in virtual reality and cognitive neuroscience for exploring the psychological aspects of physical and virtual environments. Their work summarises that a better sense of presence and immersion results in a stronger stress elicitation as human can suspend their sense of disbelief. This can be achieved by creating enough sensory congruence (consistency of senses) in both the physical and virtual environment.

In our case (outlined in Methodology lines 92-135), we generate visual and auditory stimuli in the virtual environment. The physical environment supplies the haptic feedback to give a sense of walking on a platform and elevation.

"How to define the virtual and real environment in the present study."

We have revised some of the wording in the paper to ensure that 'physical' environment is used to describe the local study set-up of the walking platform. The virtual environment is the display of the virtual world that the user operates in through the virtual reality headset.

"How to exclude the effect of experiences from real environment in the virtual environment condition?"

In this context, we mainly focus on using the virtual and physical environment to immerse the user into suspending their sense of disbelief. The main motivation for this type of stimuli is based on the

view suggested by Meehan et al. [2] that if the person has a strong sense of presence in the environment, they are more likely to generate a consistent stress response.

When designing the study, the challenge wouldn't necessarily be to exclude or remove the real-world factor. Rather, how can we give the person a strong sense of presence within the height-exposure environment to elicit a stress response? In our case, from previous literature and previous studies with this set-up, we found that this type of short-term elicitation can generate a strong and consistent stress response (observable in the ERP/ERSP).

[1] Meehan, M., Razzaque, S., Whitton, M.C. and Brooks, F.P., 2003, March. Effect of latency on presence in stressful virtual environments. In IEEE Virtual Reality, 2003. Proceedings. (pp. 141-148). IEEE.

[2] Meehan, M., Razzaque, S., Insko, B., Whitton, M. and Brooks, F.P., 2005. Review of four studies on the use of physiological reaction as a measure of presence in stressfulvirtual environments. Applied psychophysiology and biofeedback, 30, pp.239-258.

[3] Meehan, M., Insko, B., Whitton, M. and Brooks Jr, F.P., 2002. Physiological measures of presence in stressful virtual environments. Acm transactions on graphics (tog), 21(3), pp.645-652.

2. Real-world as a naturalistic environment

We've revised the paper to ensure that any "real-world" or "real world" usage refers to the naturalistic or in-the-wild environment. In this case, we describe a complex environment where the user will likely contend with more complex sensory experiences and different taskings. Our work argues that our paradigm provides a more complex sensory experience (visual, auditory, somatosensory, and haptic) and task design (walking and oddball tasks). Therefore, it is a more naturalistic stress elicitation method. This contrasts social or mental task paradigms for stress elicitation (outlined in Table 1). It could argued that these situations may appear in the real-world environment. Our view is that the traditional paradigms hold relatively low complexity compared to our design, which would be a better analogue translation study. Based on this, our analysis approach follows the MOBI (mobile brain imaging) method proposed by Klaus Gramman (one of our close colleagues), adapted for ambulatory study designs.
