# Supplementary material for: Understanding the effects of stress on the P300 response during naturalistic simulation of heights exposure
Source: PLoS One. 2024 Apr 17;19(4):e0301052. doi: 10.1371/journal.pone.0301052 (PMC11023450; doi:10.1371/journal.pone.0301052)
Supplement: Attachment — Submitted filename: PONE-D-22-20226R1_review DG_GW.docx [file pone.0301052.s003.docx]

**Manuscript Number:** PONE-D-22-20226R1 
Manuscript Title: Understanding the Effects of Physiological Stress on the P300 Response

Review by David Gallagher (incorporating comments by Grace Whittaker)

The study presented by the authors investigates the claimed relationship between physiological stress and cognitive function using a height-stressor task and a virtual reality scenario. An Oddball task was incorporated into the experimental scenario, and ERP measures taken to probe P300 response as a function of exposure to stress. Additional measures included Self Assessment Mannikin questionnaire to assess arousal level of the participant, and reaction times captured to stimuli in the oddball task. Results indicate significant differences in ratings of arousal on SAM but no significant differences based on RT or P300 ERP as a consequence of the experimental stressor exposure. Examination of data presented a basis for separating participants into two groups, differentiated by RT average between conditions – Group 1 performing worse when stressed, and Group 2 either unaffected or improving when stressed.

Principle points of criticism revolve around the definition of ‘physiological stress’ used, and the differentiation between groups based on the measures taken (including claims of a relationship between ability to cope with stress and the P300).

Firstly on the point of definition:

The title implies this pertains to ‘physiological stress’. It is assumed throughout that the experimental scenario elicits physiological stress. Yet it is not totally clear whether this does in fact elicit physiological stress (as opposed to ‘psychological stress’ or *perceived* stress). This leads to a point concerning the use of the Self Assessment Mannikin as the avowed basis for determining measures of ‘physiological stress’. Acknowledgement of the Russell-Circumplex model of valence-arousal is made, as a priori justification for using the SAM to measure (physiological) stress. Yet only the arousal component dimension of the SAM was measured, not valence and dominance. Is there a possibility that this is perhaps more indicative of ‘perceived stress’, based on an assumption here that ‘arousal’ and stress amount to the same thing. Typically when discussing physiological stress and measuring this in a behavioural study one might expect there to be more direct measures of, for instance, autonomic nervous system activity (eg. Heart rate variability). Such measures would more unequivocally determine that physiological stress is evoked within the experimental scenario. [Choi et al., (2017). Is heart rate variability (HRV) an adequate tool for evaluating human emotions? A focus on the use of the International Affective Picture System (IAPS). Psychiatry Res. 251:192-196. doi: 10.1016/j.psychres.2017.02.025. Epub 2017 Feb 11. PMID: 28213189.]

Given that such measures were not taken, at least qualifying the relationship to more directly physiological measures such as autonomic nervous system activity would provide a stronger case for using the SAM measures (and subsequent classification of individual or group differences). This includes reasoning for only focusing on the arousal dimension, not the other components of the circumplex model.

Group differentiation:

Participants were split into two groups based on their RTs, but RTs are likely influenced by a number of factors beyond stress levels, such as attention and impulsivity. Why not split participants based on SAM score or another measure of stress? The authors also claim that the experiment is aimed at investigating the relationship between the ability to cope with stress and the P300. However, the ability to cope with stress was not a variable in this study. Although the SAM results correlated with P300 amplitude across groups, when the groups were split, no effects were observed. This goes against the authors' claims that the difference between groups is a result of different abilities to cope with stress. As a suggestion, if focusing more on the relevance of SAM to unpack this (and split groups on this basis), the other dimensions of valence and dominance might usefully have contributed insight to ability to cope with the stressor. This includes understanding individual reactivity based on positive or negative valence of the experience and sense of control which may have bearing on their experience of the situation. [Jerram et al. (2013). The neural correlates of the dominance dimension of emotion. Psychiatry Res. 28;221(2):135-41. doi: 10.1016/j.pscychresns.2013.11.007. Epub 2013 Dec 4. PMID: 24359970.]

Trials with incorrect or untimely reactions were filtered out. It would be useful to know if there was a difference in the number of incorrect responses between groups. There may be a speed-accuracy tradeoff that is not accounted for when splitting the participants based on RT. How many trials were included in the analysis after incorrect trials and artifacts were removed? This can influence the EEG results obtained.

By referencing prior studies, the authors justify the election of the electrode of interest and the time window of interest for the P300. Even very small alterations in protocols can affect the timing and topography of the P300. As such, the electrode(s) and time window of interest should be defined by data-driven approaches such as PCA.

With such a small sample size, individual differences or unknown variables preclude effects (hence why P300 amplitude correlates with SAM score across participants, but not when the groups are split).

This is an interesting study that seeks to extend the capacity to assess individual reactivity to stress and neural correlates thereof in a more ecological setting. On the basis of the comments outlined, however, and concurring with previous reviewer comments this might serve best as a pilot for a more comprehensive study that addresses the points raised. It is certainly recommended that the title either be changed to remove reference to ‘physiological stress’ or indeed qualify more clearly the link to physiological bases of the stress response.
